# Supplementary material for: Analysis of chi angle distributions in free amino acids via multiplet fitting of proton scalar couplings
Source: Magn Reson (Gott). 2024 Aug 19;5(2):103–20. doi: 10.5194/mr-5-103-2024 (PMC11570886; doi:10.5194/mr-5-103-2024)
Supplement: The supplement related to this article is available online at: https://doi.org/10.5194/mr-5-103-2024-supplement. [file mr-5-103-2024-supplement.zip › Syed2024/data/fit1d_fitnmr_output.tar.gz!/fit1d/leu/900/bmse000042_simulation_2_nmredata/1H/sparse_1d.pdf]

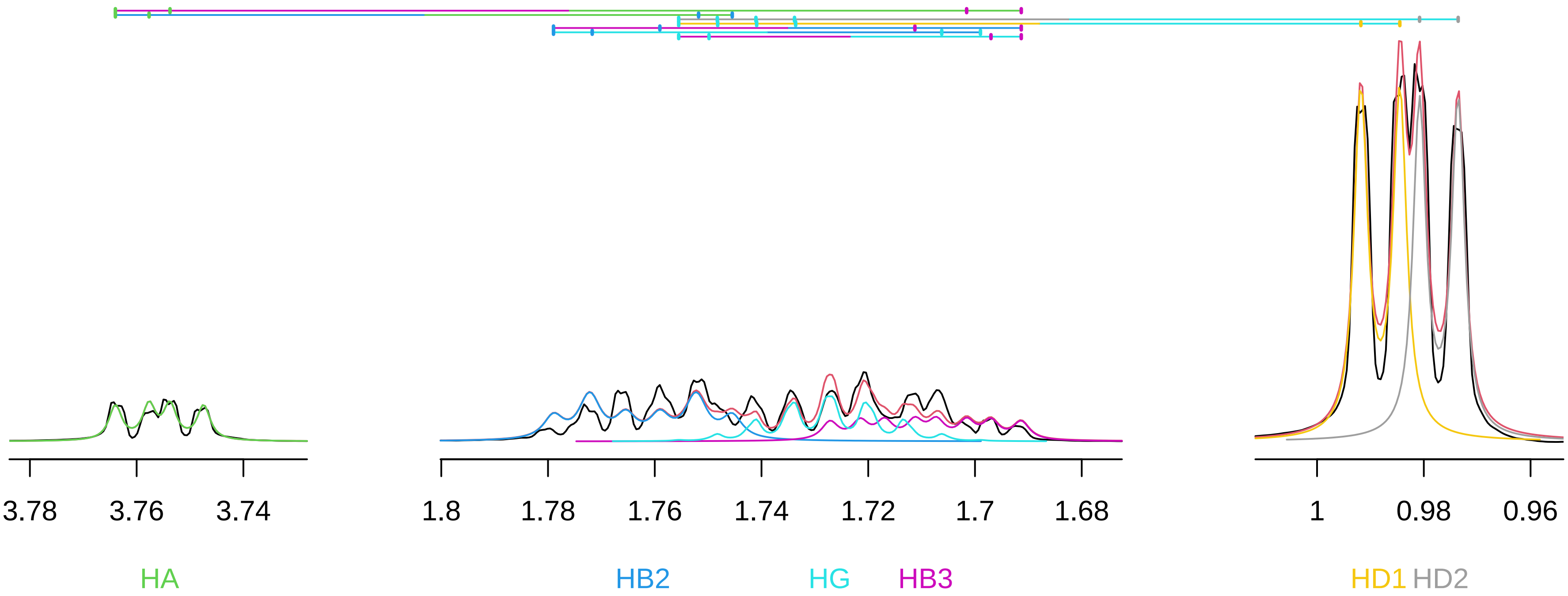

3.78
3.76
3.74
1.8
1.78
1.76
1.74
1.72
1.7
1.68
1
0.98
0.96
HA
HB2
HG
HB3
HD1
HD2
